# Supplementary material for: ANGEL2 phosphatase activity is required for non-canonical mitochondrial RNA processing
Source: Nat Commun. 2022 Sep 30;13:5750. doi: 10.1038/s41467-022-33368-9 (PMC9525292; doi:10.1038/s41467-022-33368-9)
Supplement: Supplementary file 1 — Supplementary information [file 41467_2022_33368_MOESM1_ESM.pdf]

ANGEL2 phosphatase activity is required for non-canonical mitochondrial RNA processing

Paula Clemente, Javier Calvo-Garrido, Sarah F. Pearce, Florian A. Schober, Megumi Shigematsu, Stefan J. Siira, Isabelle Laine, Henrik Spåhr, Christian Steinmetzger, Katja Petzold, Yohei Kirino, Rolf Wibom, Oliver Rackham, Aleksandra Filipovska, Joanna Rorbach, Christoph Freyer, Anna Wredenberg

Supplementary information

- Supplementary figures 1-9
- Supplementary table 1

Other Supplementary Materials for this manuscript include the following:

- Supplementary data 1. Proteomics data.
- Supplementary data 2. Oligonucleotides and Taqman probes used in this study

Supplementary Figures

a

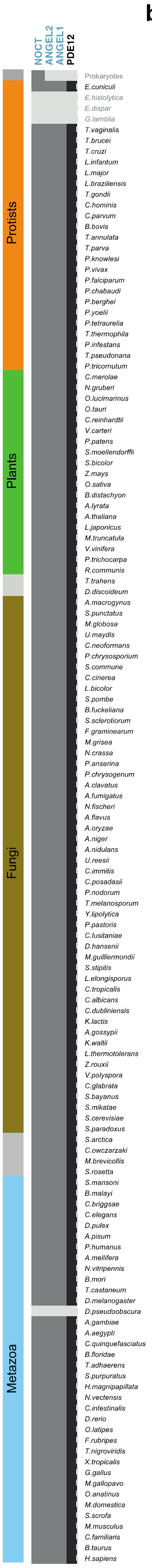

b

		Mitoprot	TargetP
Human	PDE12	0.9611	0.894
	ANGEL1	0.7899	0.458
	ANGEL2	0.6167	0.531
	Nocturnin	0.9930	0.923
	CNOT6	0.0403	0.060
	CNOT6L	0.2246	0.143
Drosophila	PDE12	0.7662	0.680
	Angel	0.9957	0.759
	Nocturnin	0.9945	0.854
	CNOT6	0.1610	0.175

c

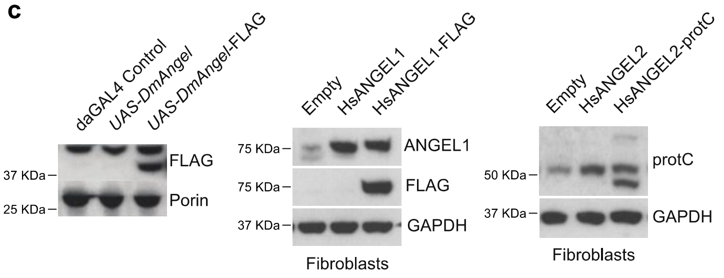

d

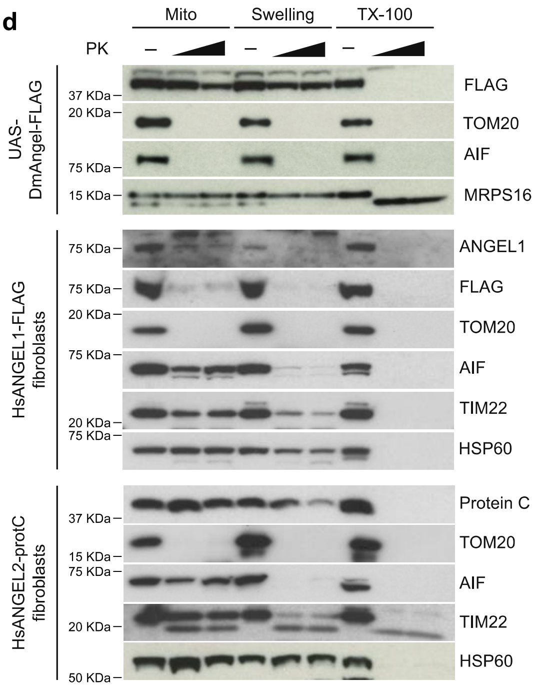

e

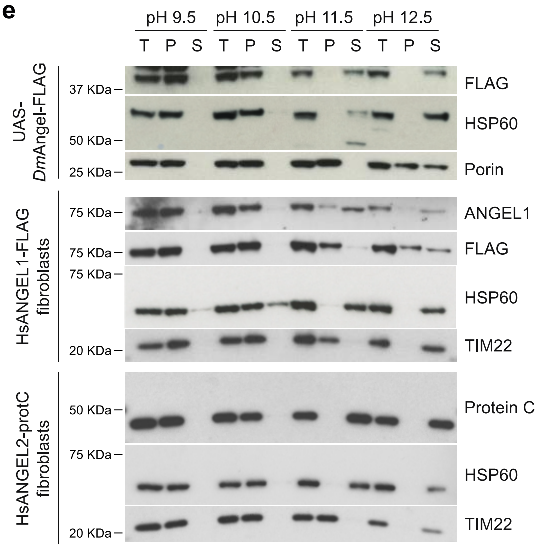

Supplementary figure 1. Identification of and subcellular localisation of carbon catabolite repression 4 (Ccr4) family members.

a Readout of co-evolutionary loss of ANGEL1, ANGEL2 and NOCTURNIN (NOCT) together with PDE12. Loss of all factors in *Drosophila pseudoobscura* is most like due to lack of annotation. **b** *In silico* prediction of subcellular localisation of various Ccr4 family members from humans or *Drosophila*, using MitoProt or TargetP. **c** Western blot analysis demonstrating overexpression of tagged *Dm*ANGEL-FLAG (left panel) in flies, or *Hs*ANGEL1-FLAG (middle) or *Hs*ANGEL2-ProtC in fibroblasts. **d** Western blot analysis of submitochondrial localisation of *Dm*ANGEL, *Hs*ANGEL1 and *Hs*ANGEL2, using samples described in (c) as determined by swelling and proteinase K (PK) treatment. Detergent Triton X-100 (TX-100) was used as control. Antibodies used as indicated. **e** Western blot analysis of submitochondrial localisation of *Dm*ANGEL, *Hs*ANGEL1 and *Hs*ANGEL2, using samples described in (c) as determined by sodium carbonate treatment at the specified pH. Antibodies used as indicated. Representative experiments are shown of 2 independent experiments performed with biologically independent samples.

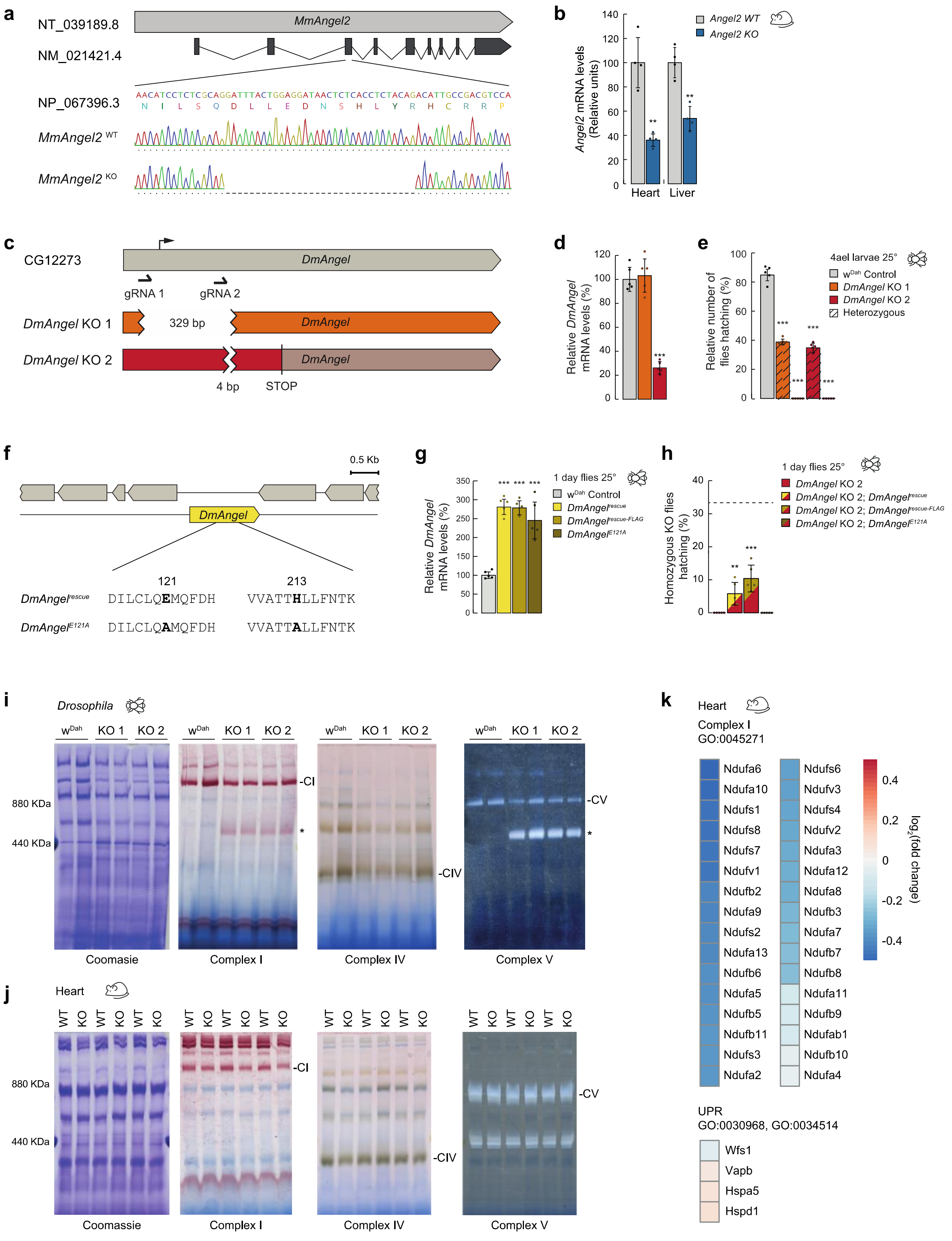

Supplementary figure 2. Knock-out of DmAngel or MmAngel2 results in an OXPHOS deficiency

a Gene (NT_039189.8) and transcript (NM_021421.4) structure of *MmAngel2* is shown. CRISPR/Cas9 editing targeted exon 3 and electropherograms of *MmAngel2*^{WT} and *MmAngel2*^{KO} genomic DNA are shown. **b** Relative *MmAngel2* transcript expression levels in control (grey) and

KO (blue) heart or liver samples at 16 weeks. (n = 4 biologically independent samples, with 3 technical repetitions). **c** Genomic arrangement of *DmAngel* (CG12273). Guide RNAs (gRNA1/2) were designed to target translation initiation. Two KO lines (orange and red) were selected. **d** Relative *DmAngel* transcript expression levels in control (wDah; grey) and *DmAngel* KO (orange and red) larvae at 4 days after egg laying. (n = 5 biologically independent samples, with 3 technical repetitions) **e** Hatching rates of control flies (wDah; grey) and flies homozygous (solid) or heterozygous (striped) for *DmAngel* KO (orange and red) (n = 5 biologically independent samples, with 2 technical repetitions). **f** Genomic constructs containing control (*DmAngel*^{rescue}), FLAG tagged (*DmAngel*$^{rescue\text{-}FLAG}$) or inactive (*DmAngel*^{E121A}) *DmAngel* alleles were used to generate rescue fly lines. **g** Relative expression levels of *DmAngel* in transgenic flies described in (f). (n=5 biologically independent samples for *DmAngel*^{rescue} or *DmAngel*^{E121A}, n = 4 biologically independent samples for *DmAngel*$^{rescue\text{-}FLAG}$ with 3 technical repetitions). **h** Hatching rates of transgenic *DmAngel*^{rescue}, *DmAngel*$^{rescue\text{-}FLAG}$, and *DmAngel*^{E121A} transgenic flies homozygous for the *DmAngel*$^{KO\ 2}$ allele (n = 5 biologically independent samples, performed once). **i** BN-PAGE and in-gel activity of control (wDah) and *DmAngel*^{KO} larvae (KO 1, KO 2) 4 days after egg laying. In-gel activities of NADH dehydrogenase (complex I, CI), cytochrome *c* oxidase (Complex IV, CIV) and ATP synthase (Complex V, CV) are shown. Partially assembled complexes of complex I and V are indicated by asterisks. (n = 2 biologically independent samples, performed once). **j** BN-PAGE and in-gel activities of 16-week-old heart mitochondria from control (WT) and *MmAngel2*^{KO} (KO) mice. (n = 3 biologically independent samples, performed once). **k** Heatmap of steady state levels of mitochondrial complex I subunits (GO: 0045271) and proteins of the unfolded protein response (GO: 0030968 and 0034514, as revealed by proteomic analysis in 16-week-old knock-out mouse hearts, relative to controls. (n = 3 biologically independent samples). All data are represented as mean ± SD with $^*p<0.05$, $^{**}p<0.01$, $^{***}p<0.001$ with 2-tailed Student's T-test.

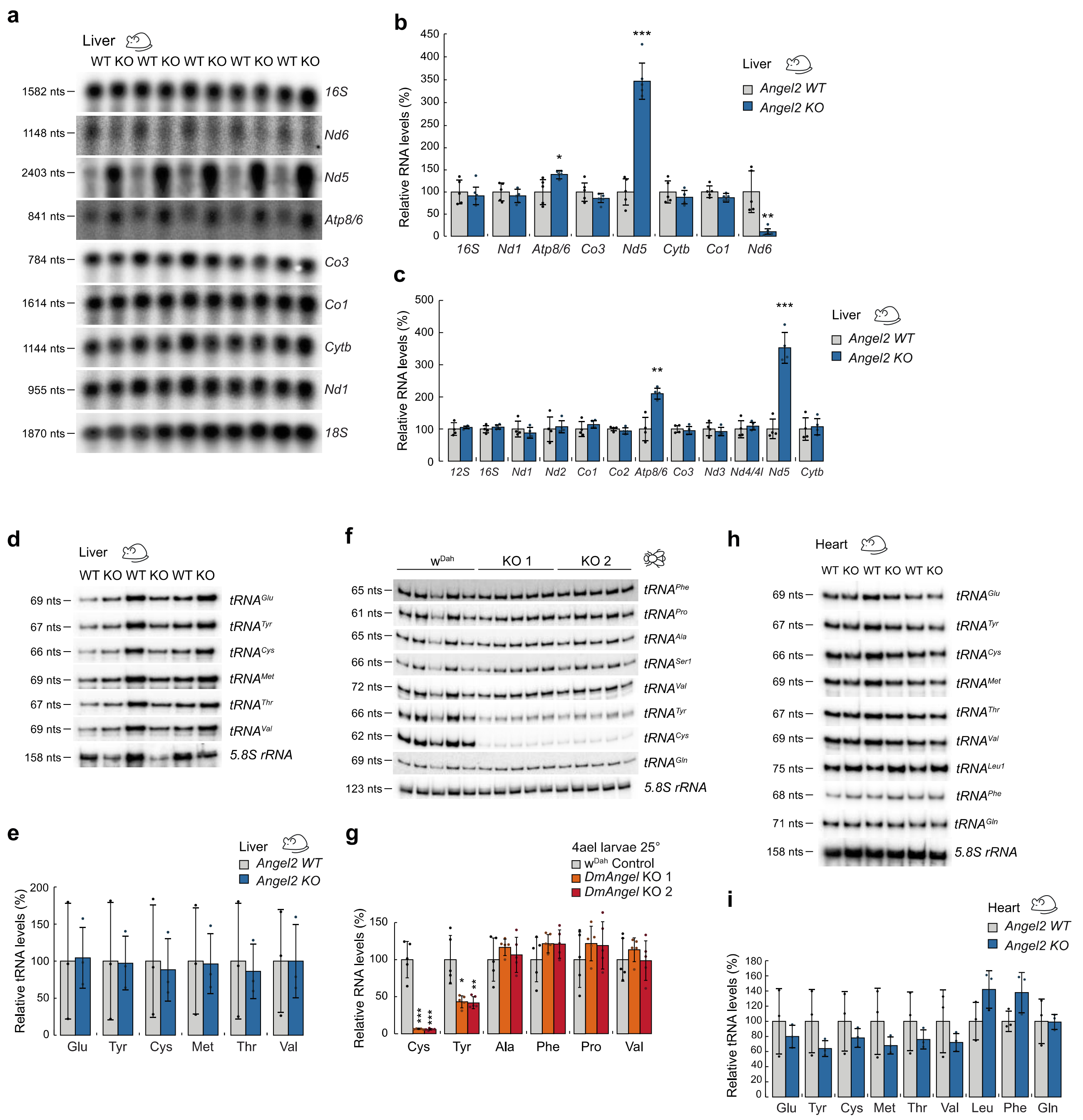

Supplementary figure 3. Knock-out of DmAngel or MmAngel2 affects mitochondrial mRNA levels

a, b, c Steady-state levels of mitochondrial transcripts in the liver of 16-week-old control (grey) or *MmAngel2*^{KO} (blue) mice as determined by (**a, b**) Northern blot (n = 5 biologically independent samples, performed once) and (**c**) qRT-PCR (n = 4 biologically independent samples, with 3 technical repetitions). 18S rRNA was used as a loading control. **d** Northern blot analysis of mitochondrial tRNAs in liver of 16-week-old control (grey) or *MmAngel2*^{KO} (blue) mice. Separation was performed by PAGE. 5.8S rRNA was used as loading control. **e** Quantification of (d). (n = 3 biologically independent samples) **f** Northern blot analysis of mitochondrial tRNAs in 4-dael control (wDah) and *DmAngel*^{KO} (KO 1, KO 2) larvae. Separation was performed by PAGE. 5.8S rRNA was used as loading control. **g** Quantification of (f) (n = 5 biologically independent samples). **h** Northern blot

analysis of mitochondrial tRNAs in the heart of 16-week-old control (grey) or *MmAngel2*^{KO} (blue) mice. 5.8S rRNA was used as loading control. **i** Quantification of (h) (n = 3 biologically independent samples). (All data are represented as mean ± SD; *p<0.05, **p<0.01, ***p<0.001 with 2-tailed Student's T-test).

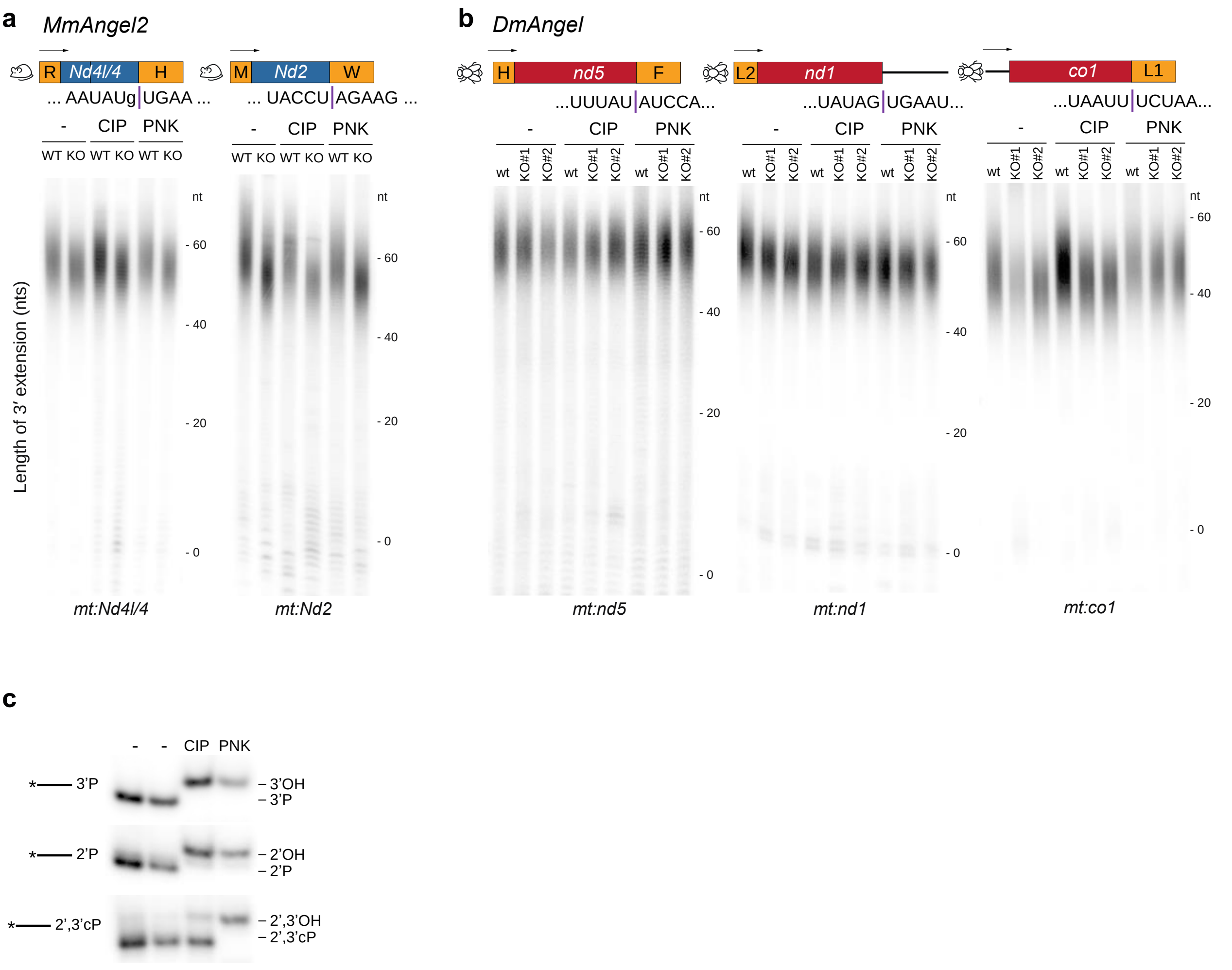

Supplementary figure 4. Mitochondrial poly(A) tail length is not affected in canonically processed transcripts in MmAngel2^{KO} mice

a *MmAngel2^{KO}*, MPAT assay on the canonical transcripts *mt:Nd4l/4* and *mt:Nd2* in mouse samples from control (WT) and *MmAngel2^{KO}* (KO) 16-week-old hearts. A representative experiment is shown of at least 3 independent experiments performed with biologically independent samples. **b** *DmAngel^{KO}*: MPAT assay on the canonical transcripts *mt:nd5* and *mt:co1*, and the non-canonical transcript *mt:nd1* in samples from control (WT) and *DmAngel^{KO}* (KO1/2, red) larvae at 4 days after egg laying. A representative experiment is shown of at least 3 independent experiments performed with biologically independent samples. **c** In vitro hydrolysis of terminal phosphates by calf intestinal phosphatase (CIP) or T4 polynucleotide kinase (PNK). 5′ end-labelled RNA oligos containing either 2′- ,3′-, or 2′,3′ cyclic phosphates were incubated with CIP or PNK, followed by PAGE separation. A representative experiment is shown of 2 independent experiments.

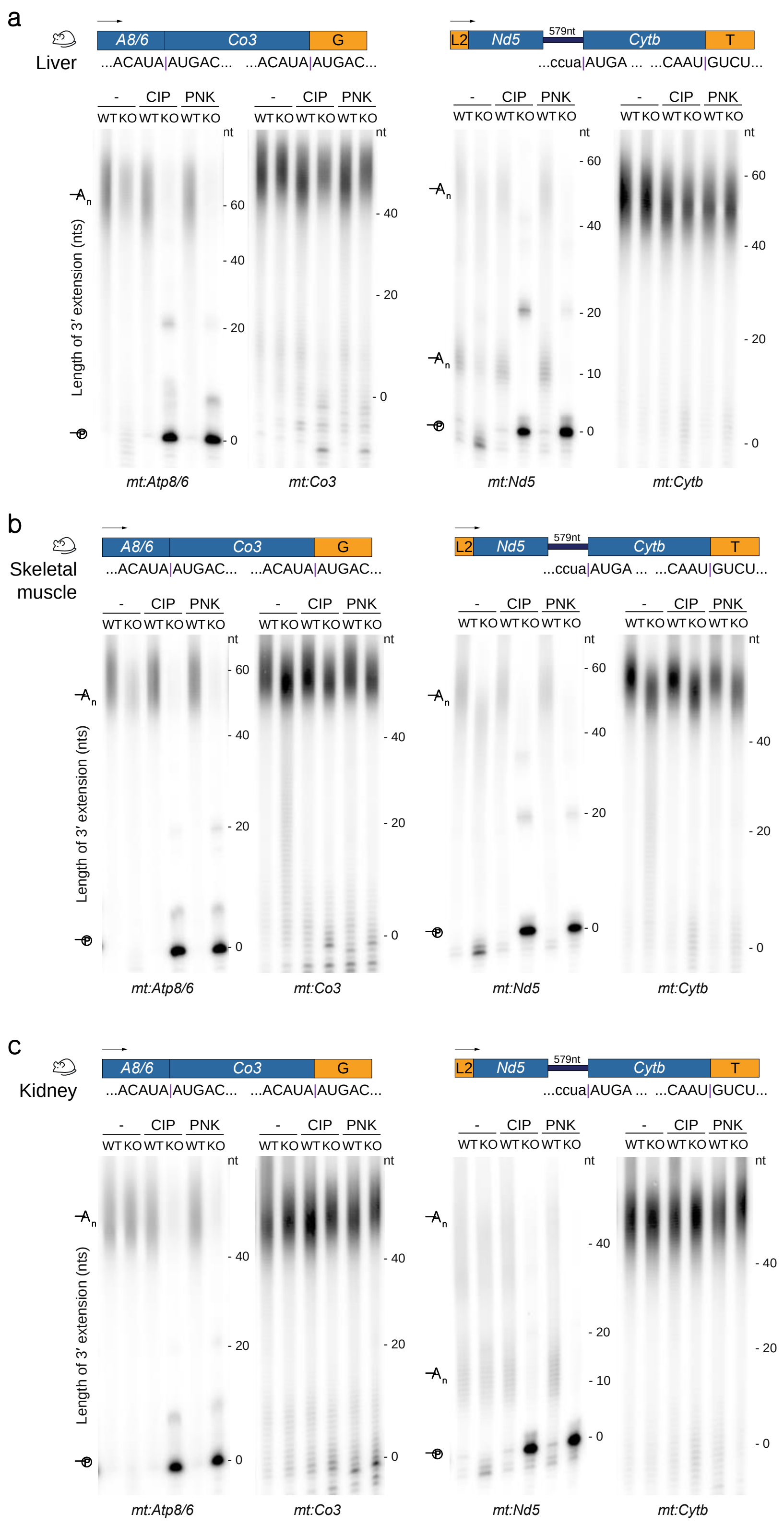

Supplementary figure 5. Loss of MmANGEL2 results in the accumulation of terminal phosphates in liver, skeletal muscle, and kidney

MPAT assay on the non-canonical transcripts *mt:Atp8/6, mt:Cox3, mt:Nd5,* and *mt:Cytb* in 16-week-old control (WT) or *MmAngel2*^{KO} (KO) in **a** liver, **b** skeletal muscle, and **c** kidney samples. Representative experiments are shown of at least 3 independent experiments performed with biologically independent samples.

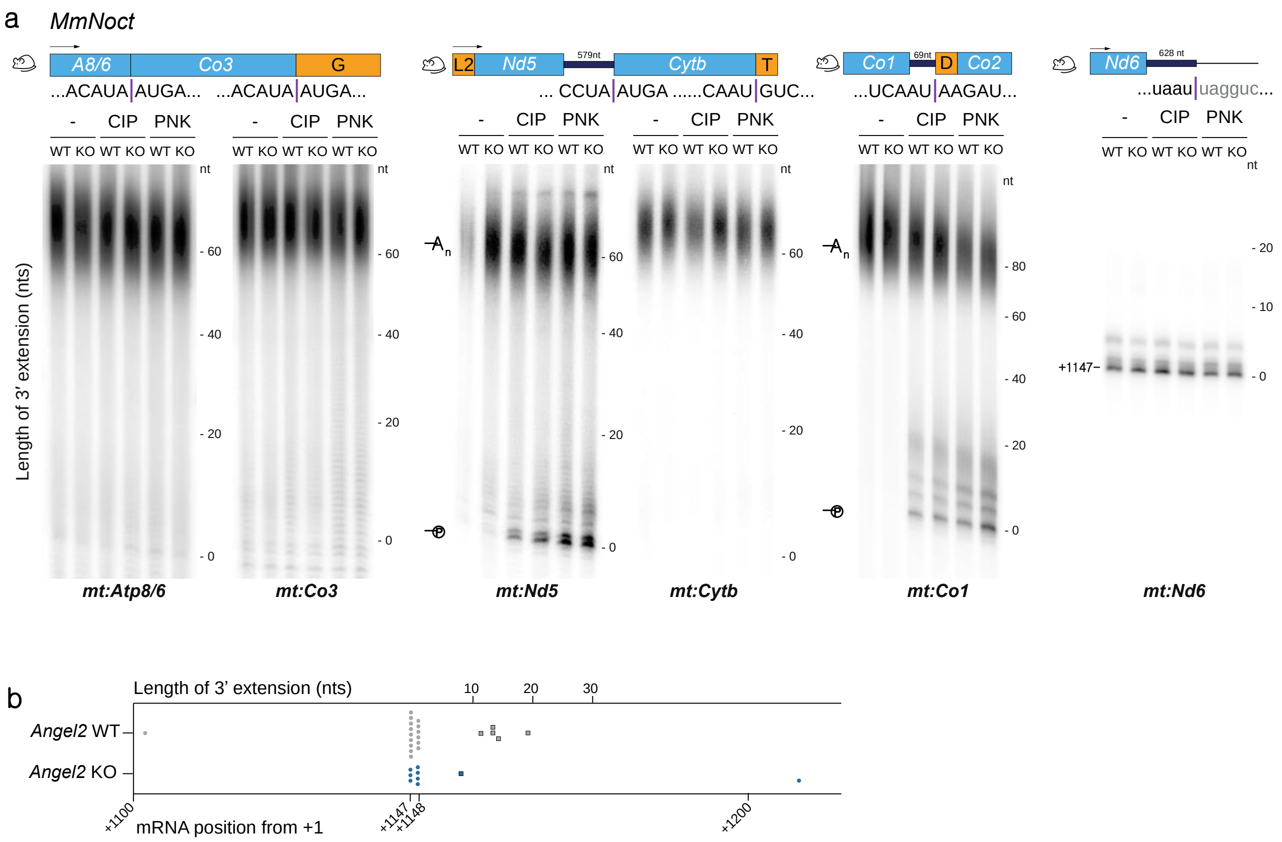

Supplementary figure 6. Mitochondrial poly(A) tail length is not affected in MmNOCT^{KO} *mice*

a *MmNoct*^{KO}: MPAT assay on the non-canonical transcripts *mt:Atp8/6*, *mt:Nd5* and *mt:Nd6*, as well as on the canonical transcripts *mt:Co3*, *mt:Cytb* and *mt:Co1* in mouse samples from control (WT) and *MmNoct*^{KO} (KO) 12-week-old hearts. CIP: calf intestinal phosphatase, hydrolyses phosphomonoester bonds. PNK: T4 polynucleotide kinase, hydrolyses phosphomonoester bonds or 2′,3′ cyclic phosphodiester from RNA ends. Single letter code of flanking tRNAs (orange) is shown. Non-coding sequences are lower case. Sequences not annotated are shown in lower case grey. Poly(A) tail length is calculated from the annotated 3′ end. Poly(A) tails (-A_n) and 3′ phosphates (-Ⓟ) are shown. The experiment was only performed once. **b** Circularisation and sequencing of *mt:Nd6* in control (WT, grey, n = 21 individual transcripts sequenced) and *MmAngel2*^{KO} (KO, blue, n = 9 individual transcripts sequenced) 16-week-old hearts. Squares represent clones that carry a poly(A) tail.

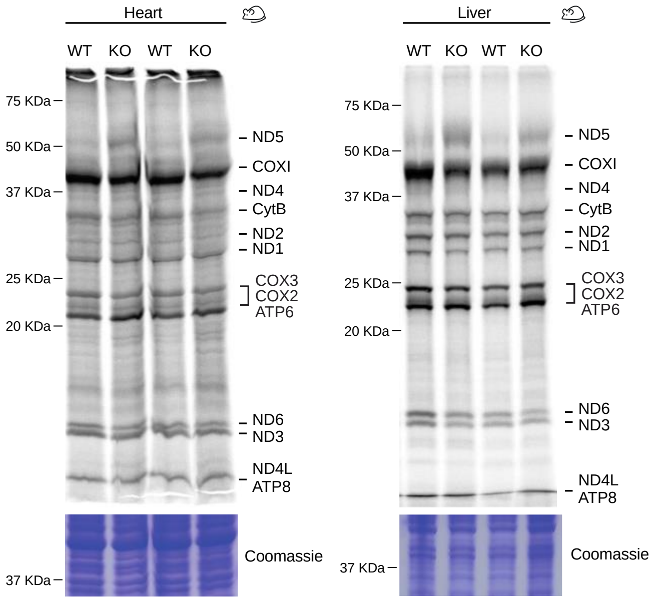

Supplementary figure 7. Loss of ANGEL2 affects mitochondrial translation

In organello translation in mitochondrial extracts from 16-week-old control (WT) and *MmAngel2*^{KO} (KO) heart and liver samples. Approximate identification of mitochondrial proteins is given. Coomassie stain is shown as loading control. A representative experiment is shown (n = 2 biologically independent samples) of 2 independent experiments performed.

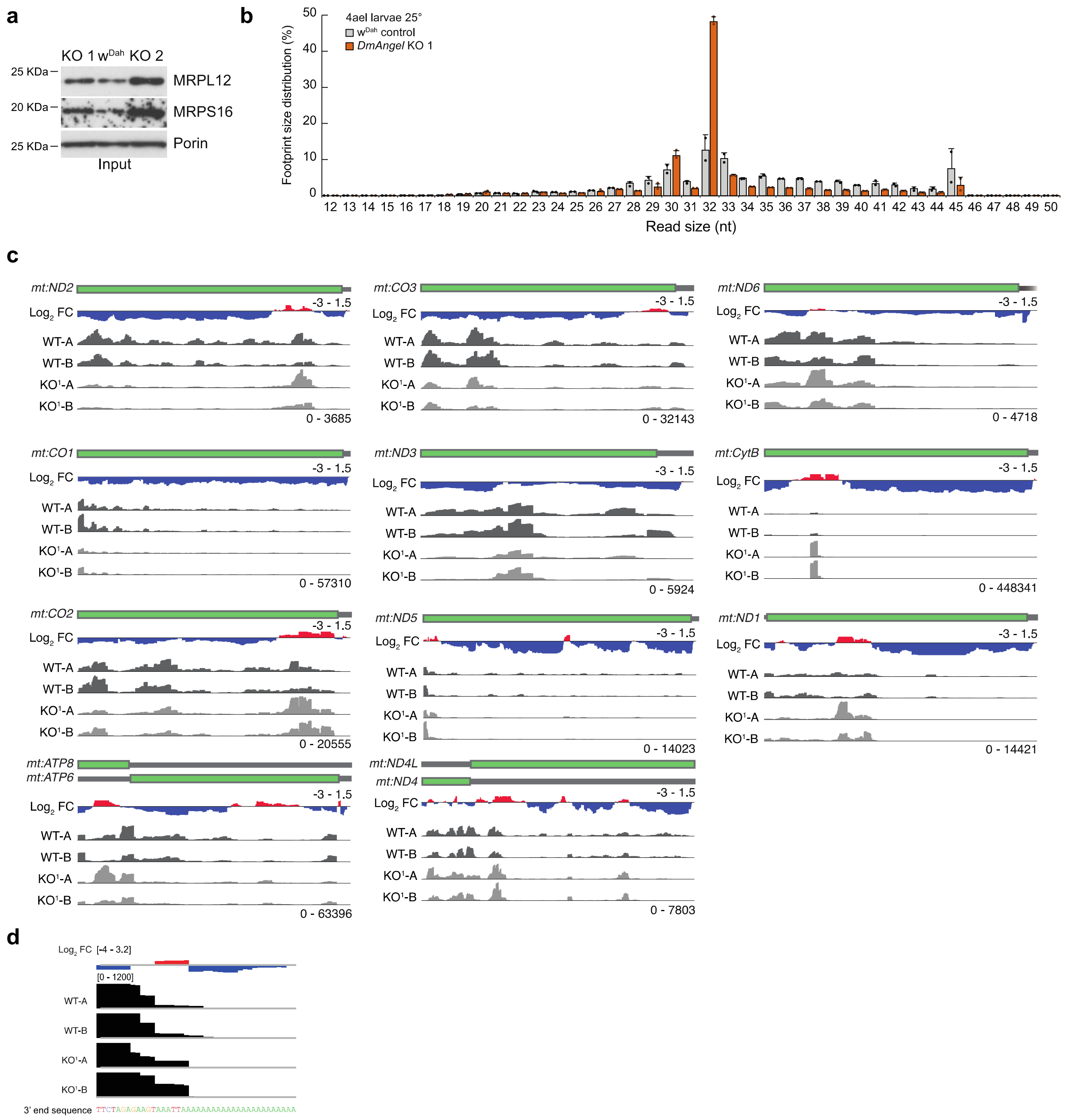

Supplementary figure 8. Loss of DmAngel affects translatome composition

a Western blot analysis of input samples used for ribosome gradients, decorated with antibodies against the small (MRPS16) and large (MRPL12) mitochondrial subunits from control (w^{Dah}) and *DmAngel*^{KO} (KO1/2) mitochondrial preparations from 4 days after egg laying (dael) larvae. A representative experiment is shown of 4 independent experiments performed with biologically independent samples. **b** Read size distribution of all mitoribosomal footprints identified in ribosome profiles of mitochondria isolated from control and *DmAngel*^{KO} larvae. (mean ± SD; *p<0.05, **p<0.01, ***p<0.001 with 2-tailed Student's T-test, n = 2 biologically independent samples) **c** Ribosome profiling showing enriched (red) or depleted (blue) footprints on mt-mRNAs in the

knockout compared to control mitochondria (log_2 fold change range from -3 – 1.5). Double tracks per control (dark grey) or angel knockout (light grey) show the read distribution across each mRNA and counts are identified at the bottom of each track. Results from two independent ribosome profiling experiments on 4 dael control (WT-A, WT-B) and *DmAngel*$^{KO-1}$ (KO^1-A, KO^1-B) larvae (mean ± SD). **d** Ribosome footprints across the 3′ end of *mt:atp6* mRNA, showing enriched (red) or depleted (blue) footprints on mt-mRNAs in the knockout compared to control mitochondria. Results from two independent ribosome profiling experiments on 4 dael control (WT-A, WT-B) and *DmAngel*$^{KO-1}$ (KO^1-A, KO^1-B) larvae.

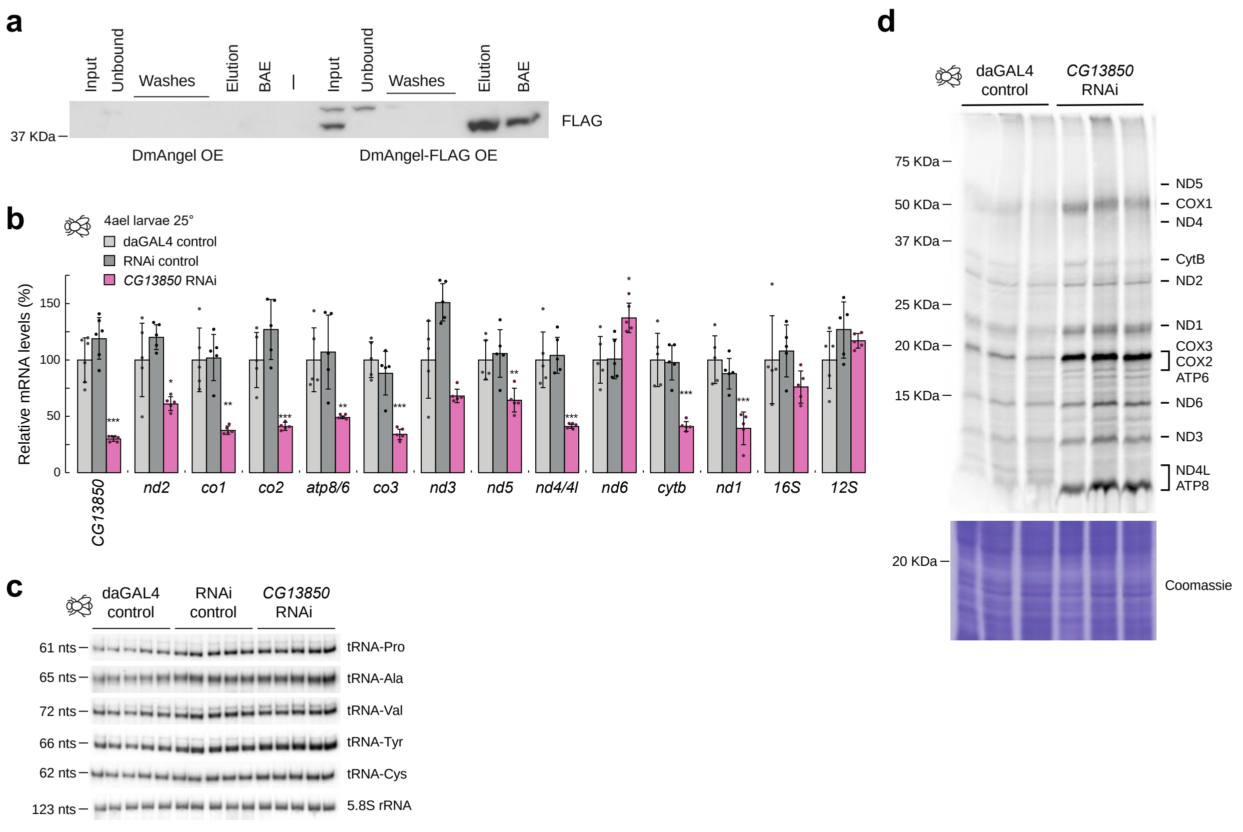

Supplementary figure 9. DmAngel interacts with CG13850, a Drosophila homolog of the mammalian FAS-activated serine/threonine kinase domain-containing protein family (FASTK).

a Western blot analysis of the fractions obtained in *Dm*Angel-FLAG immunoprecipitation (IP) experiments. A representative experiment is shown of 4 independent experiments performed with biologically independent samples. **b** Relative CG13850 and mitochondrial transcript steady-state levels in CG13850 knock-down larvae (pink; *CG13850* RNAi) and control larvae (light grey, daGAL4 control; dark grey, RNAi control) at 4-days-after-egg-laying (dael). (mean ± SD; *p<0.05, **p<0.01, ***p<0.001 with 2-tailed Student's T-test; n = 5 biologically independent samples, with 3 technical repetitions). **c** Northern blot analysis of mitochondrial tRNAs in 4 dael control (daGAL4 control, RNAi control) and *CG13850* KD larvae. Separation was performed by PAGE. 5.8S rRNA was used as loading control. (n = 5 biologically independent samples, performed once). **d** In organello translation in mitochondrial extracts from 4-dael control and *CG13850* KD larvae. Approximate identification of mitochondrial proteins is given. Coomassie stain is shown as loading control. (n = 3 biologically independent samples, performed once).

Supplementary table 1.

Fas activated serine/threonine kinase (FASTK) family orthologs and paralogs and their NCBI accession numbers.

Species	Name	NCBI RefSeq
***Danio rerio* (Zebrafish)**	FASTK	NP_001164290.1
	FASTKD1	NP_991192.2
	FASTKD2	XP_692810.5
	FASTKD3	XP_001338251.1
	FASTKD4	XP_690863.4
	FASTKD5	XP_698448.3
***Homo sapiens* (Human)**	FASTK	NP_006703.1
	FASTKD1	NP_001308975.1
	FASTKD2	NP_055744.2
	FASTKD3	NP_076996.2
	FASTKD4	NP_004740.2
	FASTKD5	NP_068598.1
***Mus musculus* (Mouse)**	FASTK	NP_075718.2
	FASTKD1	NP_796218.2
	FASTKD2	NP_766010.1
	FASTKD3	NP_001317352.1
	FASTKD4	NP_001123929.1
	FASTKD5	NP_001139556.2
***Rattus norvegicus* (Rat)**	FASTK	NP_001358218.1
	FASTKD1	NP_001178667.1
	FASTKD2	NP_001009673.1
	FASTKD3	NP_001076043.1
	FASTKD4	NP_001012154.1
	FASTKD5	XP_008760460.2
***Xenopus tropicalis* (Frog)**	FASTKD1	NP_001039188.1
	FASTKD3	NP_001135619.2
	FASTKD4	NP_001004904.2
	FASTKD5	XP_002936403.2
***Drosophila melanogaster* (fruitfly)**	CG13850	NP_651038
	CG31643	NP_723145.1
