# Supplementary material for: ANGEL2 phosphatase activity is required for non-canonical mitochondrial RNA processing
Source: Nat Commun. 2022 Sep 30;13:5750. doi: 10.1038/s41467-022-33368-9 (PMC9525292; doi:10.1038/s41467-022-33368-9)
Supplement: Supplementary file 3 — Description of additional Supplementary File [file 41467_2022_33368_MOESM3_ESM.pdf]

<u>Descriptions of additional Supplementary files</u>

Supplementary data 1. Proteomics data.

Supplementary data 2. Oligonucleotides and Taqman probes used in this study
