# Supplementary material for: ANGEL2 phosphatase activity is required for non-canonical mitochondrial RNA processing
Source: Nat Commun. 2022 Sep 30;13:5750. doi: 10.1038/s41467-022-33368-9 (PMC9525292; doi:10.1038/s41467-022-33368-9)
Supplement: Supplementary file 6 — Reporting Summary [file 41467_2022_33368_MOESM6_ESM.pdf]

nature portfolio

Corresponding author(s): Paula Clemente
Christoph Freyer
Anna Wredenberg

Last updated by author(s): Sep 6, 2022

Reporting Summary

Nature Portfolio wishes to improve the reproducibility of the work that we publish. This form provides structure for consistency and transparency in reporting. For further information on Nature Portfolio policies, see our Editorial Policies and the Editorial Policy Checklist.

Statistics

For all statistical analyses, confirm that the following items are present in the figure legend, table legend, main text, or Methods section.

n/a	Confirmed	
☐	☒	The exact sample size (*n*) for each experimental group/condition, given as a discrete number and unit of measurement
☐	☒	A statement on whether measurements were taken from distinct samples or whether the same sample was measured repeatedly
☐	☒	The statistical test(s) used AND whether they are one- or two-sided<br>*Only common tests should be described solely by name; describe more complex techniques in the Methods section.*
☒	☐	A description of all covariates tested
☒	☐	A description of any assumptions or corrections, such as tests of normality and adjustment for multiple comparisons
☐	☒	A full description of the statistical parameters including central tendency (e.g. means) or other basic estimates (e.g. regression coefficient) AND variation (e.g. standard deviation) or associated estimates of uncertainty (e.g. confidence intervals)
☐	☒	For null hypothesis testing, the test statistic (e.g. *F*, *t*, *r*) with confidence intervals, effect sizes, degrees of freedom and *P* value noted<br>*Give P values as exact values whenever suitable.*
☒	☐	For Bayesian analysis, information on the choice of priors and Markov chain Monte Carlo settings
☒	☐	For hierarchical and complex designs, identification of the appropriate level for tests and full reporting of outcomes
☒	☐	Estimates of effect sizes (e.g. Cohen's *d*, Pearson's *r*), indicating how they were calculated

Our web collection on statistics for biologists contains articles on many of the points above.

Software and code

Policy information about availability of computer code

Data collection	NextSeq Control Software 4.0.1.41/RTA 2.11.3.
Data analysis	ImageQuant TL v8.1(GE Healthcare), R v4.1.1, Proteome Discoverer v2.5 (Thermo Fisher Scientific), MaxQuant v1.6.3.4, R v4.1.1, BBTools suite v38.79, Bowtie2 v2.4.1, BEDTools81 v2.26.0.

For manuscripts utilizing custom algorithms or software that are central to the research but not yet described in published literature, software must be made available to editors and reviewers. We strongly encourage code deposition in a community repository (e.g. GitHub). See the Nature Portfolio guidelines for submitting code & software for further information.

Data

Policy information about availability of data

All manuscripts must include a data availability statement. This statement should provide the following information, where applicable:

- Accession codes, unique identifiers, or web links for publicly available datasets
- A description of any restrictions on data availability
- For clinical datasets or third party data, please ensure that the statement adheres to our policy

The ribo-profile data sets generated in this study have been deposited to GEO with accession number GSE189750. MS proteomics data generated in this study have been deposited to the ProteomeXchange Consortium via the PRIDE86 partner repository with the dataset identifier PXD033394. Datasets used in this study are MitoXplorer v2, FASTA files from UniProt (D. melanogaster in October 2019, M. musculus in September 2018) and Drosophila Ensembl v99 transcriptome sequences. Source data are provided with this paper.

Field-specific reporting

Please select the one below that is the best fit for your research. If you are not sure, read the appropriate sections before making your selection.

- [x] Life sciences
- [ ] Behavioural & social sciences
- [ ] Ecological, evolutionary & environmental sciences

For a reference copy of the document with all sections, see nature.com/documents/nr-reporting-summary-flat.pdf

Life sciences study design

All studies must disclose on these points even when the disclosure is negative.

Sample size	Statistical determination of sample size was not performed. Sample size, statistical tests and P-values are indicated in the methods section or figure legends. Non-quantitative experiments were repeated two to five different times to ensure reproducibility. The sample size (n= 3-6 animals/experimental group) in quantitative experiments is based on previous experience. The phenotypic differences between control and animals with disrupted mitochondrial function is so great, that statistical significance is already observed at small samples sizes. Furthermore, control and experimental group are isogenic and usually litter mates, reducing variability.
Data exclusions	No data were excluded from the analyses.
Replication	2-5 independent biological replicas of all experiments were performed, as detailed in the figure legends, with the exception of supplemental figure 6a that was performed once. All experiments were successfully reproduced.
Randomization	Not relevant to our study. It is not possible to use randomization, since we study animals based on their genotype. Heterozygous animals are crossed (+/-) which results in three different genotypes (+/+, +/-, -/-) of which wt (+/+) and ko (-/-) are used in experiments. Our study does not involve treatments that would require assigning animals to different experimental groups. Experimental bias is addressed by preparing wt and KO samples in parallel and using appropiate controls.
Blinding	Blinding was not used for data collection or analysis, as the same researcher collected the samples, performed the experiments and analyzed the data. Experimental and control samples were always analyzed in parallel and were treated in the same manner.

Reporting for specific materials, systems and methods

We require information from authors about some types of materials, experimental systems and methods used in many studies. Here, indicate whether each material, system or method listed is relevant to your study. If you are not sure if a list item applies to your research, read the appropriate section before selecting a response.

Materials & experimental systems

n/a	Involved in the study	
[ ]	[x]	Antibodies
[ ]	[x]	Eukaryotic cell lines
[x]	[ ]	Palaeontology and archaeology
[ ]	[x]	Animals and other organisms
[x]	[ ]	Human research participants
[x]	[ ]	Clinical data
[x]	[ ]	Dual use research of concern

Methods

n/a	Involved in the study	
[x]	[ ]	ChIP-seq
[x]	[ ]	Flow cytometry
[x]	[ ]	MRI-based neuroimaging

Antibodies

Antibodies used

Primary antibodies:
anti-DDDDK (ab1257, Abcam)
anti-Histone 3 (H0164, Merck)
anti-HSP60 (AB1-SPA-807-E, Enzo Life Sciences)
anti-Protein C (A00637, GenScript)
anti-TOM20 (13929S, Cell Signalling)
anti-TOM20 (42406, Cell Signaling)
anti-AIF (ab16501, Chemicon)
anti-MRPS16 (HPA054538, Merck)
anti-MRPL12 (HPA022853, Merck)
anti-TIM22 (14927-1-AP, Proteintech)
anti-Porin (ab14734, Abcam)
anti-GAPDH (ab8245, Abcam)
anti-ANGEL1 (sab1303061, Merck)

Secondary antibodies:
mouse anti-goat IgG-HRP (sc-2354, Santa Cruz)
anti-Rabbit IgG HRP Linked F(ab')2 (NA9340-1ML, Cytiva)
anti-Mouse IgG HRP Linked F(ab')2 Fragment (NA9310-1ML, Cytiva)
Goat anti-Rabbit IgG (H+L) antibody Alexa Fluor 568 (A-11036, Thermo Fisher)
Goat anti-Mouse IgG (H+L) antibody Alexa Fluor 568 (A-11031, Thermo Fisher)

Validation

The anti-ANGEL1 antibody (Merck, https://www.sigmaaldrich.com/SE/en/product/sigma/sab1303061) has been validated in this study. The antibody recognised a 75 KDa band in human fibroblasts that overexpress human ANGEL1 or ANGEL1-FLAG. The same band is detected with anti-ANGEL1 and anti-DDDDK antibodies. The anti-ANGEL1 does not recognize the endogenous protein.

The remaining antibodies have been validated by the manufacturer, validation details and relevant publications are detailed in their respective websites.
anti-DDDDK: Abcam, https://www.abcam.com/ddddk-tag-binds-to-flag-tag-sequence-antibody-ab1257.html#description_references
anti-Histone3: Merck, https://www.sigmaaldrich.com/SE/en/product/sigma/h0164
anti-HSP60: Enzo Life Sciences, https://www.enzolifesciences.com/ADI-SPA-807/hsp60-monoclonal-antibody-lk-2/
anti-ProteinC: GenScript, https://www.genscript.com/antibody/A00637-Protein_C_Tag_Antibody_HPC4_pAb_Rabbit.html
anti-TOM20: Cell signaling, https://www.cellsignal.com/products/primary-antibodies/tom20-antibody/13929 and https://www.cellsignal.com/products/primary-antibodies/tom20-d8t4n-rabbit-mab/42406
anti-AIF: Chemicon/Merck, https://www.sigmaaldrich.com/SE/en/product/mm/ab16501
anti-MRPS16: The Human Protein Atlas/Merck, https://www.sigmaaldrich.com/SE/en/product/sigma/hpa054538 and https://www.proteinatlas.org/ENSG00000182180-MRPS16/antibody
anti-MRPL12: The Human Protein Atlas/Merck https://www.sigmaaldrich.com/SE/en/product/sigma/hpa022853 and https://www.proteinatlas.org/ENSG00000262814-MRPL12/antibody
anti-TIM22: Proteintech/Thermo Fisher, https://www.thermofisher.com/antibody/product/TIM22-Antibody-Polyclonal/14927-1-AP
anti-Porin: Abcam, https://www.abcam.com/vdac1porin-antibody-20b12af2-ab14734.html
anti-GAPDH: Abcam, https://www.abcam.com/gapdh-antibody-6c5-loading-control-ab8245.html
mouse anti-goat IgG-HRP: Santa Cruz, https://www.scbt.com/p/mouse-anti-goat-igg-hrp
anti-Rabbit IgG HRP Linked F(ab')2 (NA9340-1ML, Cytiva): Cytiva, https://www.cytivalifesciences.com/en/us/shop/protein-analysis/blotting-and-detection/blotting-standards-and-reagents/amersham-ecl-hrp-conjugated-antibodies-p-06260#productsupport
Goat anti-Rabbit IgG (H+L) antibody Alexa Fluor 568: Thermo Fisher, https://www.thermofisher.com/antibody/product/Goat-anti-Rabbit-IgG-H-L-Highly-Cross-Adsorbed-Secondary-Antibody-Polyclonal/A-11036
Goat anti-Mouse IgG (H+L) antibody Alexa Fluor 568: Thermo Fisher, https://www.thermofisher.com/antibody/product/Goat-anti-Mouse-IgG-H-L-Highly-Cross-Adsorbed-Secondary-Antibody-Polyclonal/A-11031

Eukaryotic cell lines

Policy information about cell lines

Cell line source(s)

Human fibroblast lines were obtained from the Center of inherited metabolic diseases at Karolinska University Hospital.
A control cell line with normal mitochondrial biochemistry was selected for this study. This cell line had been previously used as a control in Calvo-Garrido, J. et al. (https://www.sciencedirect.com/science/article/pii/S2213671119300256?via%3Dihub#mmc1) and was immortalised by exogenous expression of hTERT.
Phoenix-AMPHO cells were obtained from ATCC.

Authentication

Human fibroblast lines are not authenticated. Fibroblast biopsies and establishment of fibroblast cultures is part of clinical routine at the Center of inherited metabolic diseases at the Karolinska University hospital for more than 30 years. The procedure is described in the following publication: https://ng.neurology.org/content/7/2/e566.
Authentication of Phoenix-AMPHO cells was performed by ATCC https://www.atcc.org/products/crl-3213.

Mycoplasma contamination

All cell lines tested negative for mycoplasma infection

Commonly misidentified lines
(See ICLAC register)

No commonly misidentified cell lines were used in this study

Animals and other organisms

Policy information about studies involving animals; ARRIVE guidelines recommended for reporting animal research

Laboratory animals

Angel2 knock-out and wild type mice used in this study belong to strain C57BL/6N, experiments were always performed in 16-week old males and females with consistent results. In each experiment only male or female mice were used. Angel2 knock-out mice were obtained from Nanjing Biomedical Research Institute, Nanjing University, China (Strain number: T001889; B6/N-Angel2em1Cd32/Nju) and backcrossed to the C57BL/6N strain (Charles River Laboratories). Mice were house in standard individually ventilated cages with a 12-hour:12-hour light:dark cycle under controlled environmental conditions.

All Drosophila melanogaster lines in this study have been backcrossed to the Wolbacchia-free white Dahomey background. Experiments were performed on third instar larvae or one-day flies as indicated. When experiments were performed on adult flies, only males were used. The DmAngel knock out line, DmAngel rescue lines, DmAngel overexpression lines and CG13850 overexpression lines were generated in this study, as indicated in the methods section, and backcrossed to the white Dahomey genetic background. The CG13850 RNAi line was obtained from Vienna Drosophila Resource Center (VDRC line #15233) and backcrossed to to the white Dahomey genetic background. The daughterlessGAL4 line was a kind gift of Prof. Linda Partridge and was

	backcrossed to the white Dahomey genetic background.
Wild animals	The study did not involve wild animals
Field-collected samples	The study did not involve samples collected from the field
Ethics oversight	All animal procedures were approved by the Stockholm's Animal Experimentation Ethics Board (Ethical permit number 14460/18) of the Swedish Board of Agriculture and were performed in accordance with national and European law.

Note that full information on the approval of the study protocol must also be provided in the manuscript.
